# Evaluation of Kubuqi Desert Wind Erosion Prevention Service and drivers of the actual wind erosion studies Based on RWEQ Model from 2000 to 2022

**DOI:** 10.1371/journal.pone.0321260

**Published:** 2025-05-06

**Authors:** Yinchao Chai, Hejun Zuo, Min Yan, Tao Zuo, Yu Yan

**Affiliations:** 1 Inner Mongolia Key Laboratory of Aeolian Physics and Desertification Control Engineering, College of Desert Control Science and Engineering, Inner Mongolia Agricultural University, Hohhot, People’s Republic of China,; 2 Hanggin Desert Ecosystem Positioning Research Station, Hohhot, China; Ardakan University, ISLAMIC REPUBLIC OF IRAN

## Abstract

Ecosystem service research is essential to identify the contribution of the ecosystem to human welfare. As an important ecological barrier zone, the Kubuqi Desert supports the use of a crucial wind erosion prevention service (WEPS) to improve the ecological environment quality. Based on the Revised Wind Erosion Equation (RWEQ) model, the spatial and temporal changes of WEPS in the Kubuqi Desert region were simulated from 2000 to 2022, and the impacts and interactions of natural and socio-economic factors including numerical and typological variables on the spatial pattern of wind and sand control services in the region were analyzed by using geographical detector. From 2000 to 2022, the total WEPS provided in the Kubuqi Desert ranged from 0.35 × 10^7^ t to 1.26 × 10^7^ t. The average WEPS per unit area was between 0.19 kg m^-2^ to 0.68 kg m^-2^.WEPS has a higher spatial distribution in the east and a lower spatial distribution in the west. Soil type was the most important driver of the actual wind erosion (SL), with vegetation cover, elevation, mean annual temperature, mean annual wind speed, and mean annual precipitation as the main drivers, and population size and GDP as secondary drivers. The interaction analysis showed that the interaction of weather factor, vegetation factor and soil factor is the dominant factor influencing the amount of soil wind erosion in the Kubuqi Desert.

## Introduction

In dryland ecosystems, low precipitation and sparse vegetation critically compromise surface resistance to accelerated aeolian processes, resulting in land degradation marked by desiccated, coarse-textured soils with diminished fertility. Accurate prediction of aeolian soil displacement is critical for mitigating land degradation and protecting socioeconomic assets, necessitating the development of process-based aeolian transport models that quantify erosion magnitude and spatiotemporal patterns [1]. Various validated wind erosion models are currently available [2], including the Wind Erosion Equation (WEQ) model [3], the Texas Tech Erosion Analysis Model (TEAM) [4], the Wind Erosion Stochastic Simulator (WESS) [5], the Wind Erosion Prediction System (WEPS) [6], the Wind Erosion Assessment Model (WEAM) [7], and the Revised Wind Erosion Equation (RWEQ) model [8]. The model mentioned above represents equations that describe various factors influencing wind erosion processes. It is primarily used to calculate soil loss from cultivated land and can also be applied to predict soil erosion in sandy areas. Compared with other models, although the RWEQ (Revised Wind Erosion Equation) model simplifies the complexity of wind erosion, it still comprehensively considers the impacts of climate, soil properties, and vegetation cover on wind erosion. Consequently, its application in multi-scale wind erosion simulations has garnered significant attention. It has been successfully implemented in northern China [9–11] due to its effectiveness in simulating the windbreak and sand fixation functions of vegetation in this region [12]. Additionally, the RWEQ model has proven capable of accurately calculating wind-induced soil erosion in cultivated lands in Inner Mongolia [13]. In summary, this study selects the RWEQ model as the core methodology to predict wind erosion quantities in the Kubuqi Desert.

The Kubuqi Desert, situated in the western region of Inner Mongolia, which is particularly susceptible to wind erosion compared to the eastern parts of the country [14]. It encompasses a variety of landforms, including mobile dunes, semi-fixed dunes, fixed dunes, lakeshore terraces, wetlands, and lawn wetlands [15]. The desert spans northward to Bayannur City, across the Yellow River, and is characterized by its expansive grasslands and diverse natural environmental conditions. The Kubuqi Desert ecosystem stands out as one of the most emblematic and representative examples of China’s arid and semi-arid zones [16], rendering it particularly significant for research in this area. Given the diverse surface types within the Kubuqi Desert, WEPS are crucial. However, there is a scarcity of research identifying which factors, whether natural or social, predominantly influence WEPS in this region. Relevant studies have shown that in northern China, the coupled effects of temperature, precipitation and wind speed are the main climatic drivers of sand fixation function [17]. In addition, land use changes such as farmland expansion and urbanisation, as well as anthropogenic factors such as grazing, can lead to the reduction of WEPS to some extent [18–19]. Li used bifurcated pixel model [20], intensity analysis and residual analysis to analyse vegetation cover changes in Inner Mongolia, and vegetation cover changes were explored in relation to climate and human activities, and the results showed that the increase in afforestation area is the main factor driving the improvement of WEPS in Inner Mongolia. Dang investigated the impact of land use on soil wind erosion in the Kubuqi Desert using multi-source data [21] and integrating object-oriented, decision tree, and assisted human-computer interaction interpretation methods, and concluded that the sand quality change contributes the most to the reduction of the intensity of soil wind erosion in the region. Current studies clarify WEPS spatial drivers but inadequately address factor interactions. Prevailing single-factor analyses lack comprehensive assessment of multi-driver synergies.

Geodetector is a statistical method grounded in spatial differentiation, designed to uncover the driving mechanisms behind variables [22]. This methodology has gained widespread adoption across interdisciplinary research domains, particularly in ecology, meteorology, and regional planning [23–25], where it effectively disentangles complex spatial-temporal dynamics through rigorous detection of stratified heterogeneity mechanisms. Nonetheless, the use of geoprobes to examine the factors influencing WEPS (Wind Erosion Prediction System) is relatively sparse. A limited number of studies have conducted exploratory research on the drivers of WEPS system changes in the Xilin Gol League using geoprobes [26]. These studies have demonstrated that the application of geoprobe analysis in WEPS is both reasonable and scientifically sound [27]. Given that the accuracy of the RWEQ model has not been field-validated in the Kubuqi Desert, this paper attempts to validate its accuracy based on field-acquired measured sand transport data, and simulates the spatial and temporal changes of WEPS in the Kubuqi Desert from 2000 to 2022. The focus is to address the following scientific questions: how the wind erosion condition of the Kubuqi Desert varies in time and space from 2000 to 2022, to evaluate the WEPS and to identify its drivers with the help of the GeoFactor Probe. This study will improve the understanding of the spatial pattern of WEPS in the Kubuqi Desert and can provide recommendations for governmental policies on ecological management.

## Materials and methods

### Study Area

**Fig 1:**
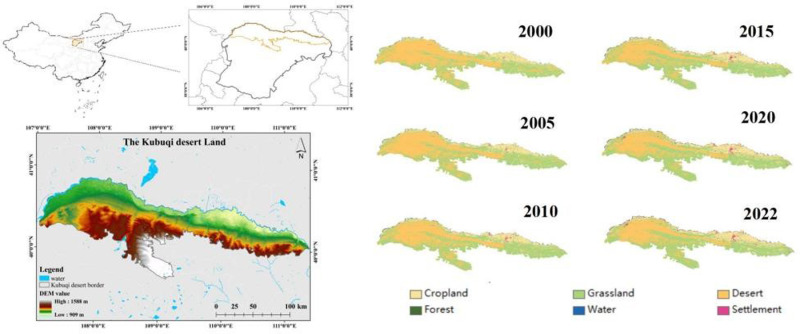
Location of the Kubuqi Desert and the land cover map from 2000 to 2022.

The Kubuqi Desert is the seventh largest desert in China, located in the southern part of the Hetao Basin on the south bank of the Yellow River (39°15′-40°45′N, 107°00′-111°30′E), covering an area of about 1.86 × 10^4^ km^2^ [9], and administratively belonging to Ordos City, Inner Mongolia Autonomous Region of China (Fig 1), including three banners: Hangjin Banner, Dalat Banner, and Jungar Banner, with elevations ranging from 909 to 1589 m above sea level, which is one of the large deserts near the Loess Plateau. The climate type of the region is temperate continental and the soil type is dominated by sandy soil.

## Methods and data

### Calculation of five factors

The RWEQ model is an empirical tool grounded in extensive field experiments. It offers a foundation for the prevention and management of land degradation by estimating wind erosion of regional soils over long time periods with high spatial and temporal precision. The model’s core principle involves considering wind erosion in the absence of vegetation as the potential erosion. In contrast, wind erosion occurring with vegetation cover represents the actual erosion. The model quantifies the efficacy of wind erosion prevention and control services by calculating the difference between potential and actual wind erosion. The calculation formula is as follows:


WEPS=SLR−SL (1)

where WEPS is the amount of wind erosion prevention service (kg/m²); SLR is the potential wind erosion amount (kg/m²); and SL is the actual wind erosion amount (kg/m²).The potential wind erosion amount SLR is calculated using the following formula:


$$\text{SLR}=\frac{2z}{{\text{sr}}^{\text{2}}}{\text{Qrmaxe}}^{-{\left(\frac{\text{z}}{\text{sr}}\right)}^{\text{2}}}$$ (2)

$$\text{sr}=\text{150}.71\times {\left(\text{WF}\times \text{EF}\times \text{SCF}\times {\text{K}}^{'}\right)}^{-\text{0}.3711}$$ (3)

$$\text{Qrmax}=\text{109}.8\times \left(\text{WF}\times \text{EF}\times \text{SCF}\times {\text{K}}^{'}\right)$$ (4)

where SLR is the potential soil wind erosion amount (kg/m^2^); Qr max is the maximum wind sand transfer amount (kg/m); S is the length of the key plot (m); z is the maximum wind erosion occurrence distance in the downwind direction (m); WF is the climate factor (kg/m); K’ is the surface roughness factor; EF is the soil erodibility factor; SCF is the soil crusting factor; and C is the vegetation cover factor.

The actual wind erosion SL was calculated as follows:


$$\text{SL}=\frac{\text{2z}}{{\text{S}}^{2}}{\text{Qmaxe}}^{-{\left(\frac{\text{z}}{s}\right)}^{\text{2}}}$$ (5)

$$\text{S}=\text{150}.71\times {\left(\text{WF}\times \text{EF}\times \text{SCF}\times {\text{K}}^{'}\times \text{C}\right)}^{-\text{0}.3711}$$ (6)

$$\text{Qmax}=\text{109}.8\times \left(\text{WF}\times \text{EF}\times \text{SCF}\times {\text{K}}^{'}\times \text{C}\right)$$ (7)

where SL is the actual soil wind erosion (kg/m^2^); Qmax is the maximum wind sand transfer (kg/m); S is the length of the critical plot (m); z is the distance of maximum wind erosion occurrence in the downwind direction (m); WF is the climate factor (kg/m); K’ is the surface roughness factor; EF is the soil erodibility factor; SCF is the soil crust factor; and C is the vegetation cover factor.

The following are the extraction equations for each input factor of the RWEQ model:

#### Weather factor.

Weather conditions such as wind speed, temperature, rainfall, solar radiation and snow cover days all affect the soil wind erosion modulus and together they form the climate factor. Climate factor WF characterises the ability of wind to transport soil particles, taking into account rainfall, temperature, insolation and snow cover, and is calculated as follows.


$$\text{Wf}=\text{u2}\times {\left(\text{u2}-\text{u1}\right)}^{\text{2}}\times \text{Nd}$$ (8)

$$\text{ρ}=\text{348}.01\left(\frac{\text{1}.013-\text{0}.1183\times \text{EL}+\text{0}.0048\times {\text{EL}}^{\text{2}}}{\text{T}}\right)$$ (9)

$$\text{SW}=\frac{\text{ETp}-\left(\text{R}+\text{I}\right)\times \frac{\text{Rd}}{\text{Nd}}}{\text{ETp}}$$ (10)

$$\text{SD}=\text{1}-\text{P}\left(\text{Hsnow}>\text{25}.4mm\right)$$ (11)

$$\text{WF}=\text{Wf}\times \frac{\text{ρ}}{g}\times \text{SW}\times \text{SD}$$ (12)

where WF is the meteorological factor (kg/m); Wf is the wind field intensity factor (m^3^/s^3^), which is calculated according to the wind field intensity factor method, and is obtained from the monthly average wind speed µ_2_ (m/s) at 2m of the monitoring point, the sanding wind speed µ_1_ (assumed to be 5m/s), and the number of days Nd with daily average wind speeds greater than 5m/s during the observation cycle; ρ is the air density (kg/m^3^), which is calculated from the altitude EL (km) and absolute temperature T (K): g is the acceleration of gravity (usually 9.8 m/s^2^); SW is the monthly multi-year average soil moisture factor (dimensionless); R is the amount of rainfall (mm); I is the amount of irrigation (mm), China’s meteorological database does not include values such as I, so I will be set to 0; Rd is the number of times of rainfall and/or the number of days of irrigation; ETP is the potential relative surface evapotranspiration (mm), calculated by the number of days Nd during the observation cycle. evapotranspiration (mm), which is calculated from solar radiation SR (cal/cm^2^) and average temperature DT (°C); SD is the snow cover factor (dimensionless); and P is the probability that the snow cover depth (Hsnow) is greater than 25.4 mm in the calculated time period.

In this study, wind speed and rainfall data were spatially interpolated and analyzed using the Inverse Distance Weighted (IDW) method, based on the distribution of meteorological stations within the study area. This approach yielded meteorological data with spatial continuity. Furthermore, the RWEQ model necessitates wind speed data to be at a height of 2 meters above the ground. However, the wind speed data collected by the automatic meteorological observation stations used in this research were recorded at a height of 10 meters. It is well-known that wind speeds vary with elevation; the friction at the soil surface decelerates wind, resulting in the slowest speeds at ground level, which then increase with height. Meteorological science typically standardizes anemometer placement at a height of 10 meters, whereas agrometeorological science often requires anemometers to be positioned at 2 or 3 meters to facilitate evapotranspiration calculations. To address this discrepancy, we applied the wind speed conversion standards issued by the Food and Agriculture Organization of the United Nations (FAO) to adjust the 10-meter wind speed measurements to a 2-meter height. The conversion formulas are detailed below:


$$\text{U2}=\text{Uz}\frac{\text{4}.87}{\text{ln(67}.8z-5\text{.42)}}$$ (13)

where U_2_ represents the wind speed at 2 metres and z represents the height to be converted, which is taken as 10m in this paper.

#### Erodible factor.

In this study, we assumed that the EF and SCF factors did not change over time. Erodibility factor refers to the sensitivity of the soil to erosion, for different mechanical composition and physicochemical properties of soil types, the smaller the particle size and the lower the content of organic matter, the greater its soil erodibility, the more prone to erosion; conversely, the coarser the particle size and the higher the content of organic matter, the smaller its erodibility, the less prone to erosion. The formula for the upper soil erodibility factor is as follows.


$$\text{EF}=\frac{\text{29}.9+\text{0}.31Sa+\text{0}.17Si+\text{0}.33\left(\frac{\text{Sa}}{\text{Cl}}\right)-\text{2}.59OM-\text{0}.95CaCO3}{\text{100}}$$ (14)

where EF is the soil erodibility factor; Sa is the soil coarse sand content in %; Si is the soil silt content in %; Cl is the soil clay content in %; OM is the soil organic matter content in %; CaCO3 is the soil calcium carbonate content in %.

#### Soil crust factor.

Soil crust refers to the micro-layers formed by the interaction of certain lower organisms with the soil surface or by precipitation drops on the soil surface, and is generally classified into biological crust and physical crust according to the mechanism of production. Biological crusts are favourable to the resistance of soil to wind erosion: physical crusts are fragile and, on the contrary, accelerate the process of soil erosion by wind, as calculated by the following formula.


$$\text{SCF}=\frac{\text{1}}{\text{1}+\text{0}{\text{.0066Cl}}^{\text{2}}+\text{0}{\text{.021OM}}^{\text{2}}}$$ (15)

where SCF is the soil crust factor; Cl is the soil clay content in %; OM is the soil organic matter content in %.

#### Vegetation factor.

Different vegetation types have different ability to prevent wind and fix sand according to their ecological characteristics, and the vegetation cover factor in the RWEQ model indicates the magnitude of inhibition of soil wind erosion under a certain vegetation cover condition.


$$\text{C}={\text{e}}^{\text{ai}\left(\text{sc}\right)}$$ (16)

$$\text{SC}=\frac{\text{NDVI}-\text{NDVIsoil}}{\text{NDVIveg}-\text{NDVIsoil}}$$ (17)

where C is the vegetation cover factor; SC is the vegetation cover with a scale of 1, calculated from the NDVI dataset; ai is taken as -0.0438 in this paper; NDVI is the Normalised Vegetation Index (NDVI), NDVIsoil is the NDVI value of bare soil pixels, and is taken as the NDVI value corresponding to a confidence level of 5%, and NDVIveg is the NDVI value of the largest vegetation, and is taken as the NDVI value corresponding to a confidence level of 95%. NDVI value corresponding to 95% confidence level.

#### Tillage roughness.

Surface roughness is the effect of land surface roughness caused by topography on soil wind erosion and is calculated by the following formula:


$${\text{K}}^{\text{'}}={\text{e}}^{\left(\text{1}.86Kr-\text{2}{\text{.41Kr}}^{\text{0}.94}-\text{0}.127Crr\right)}$$ (18)

$$\text{Kr}=\text{0}.2\times \frac{{\left(\text{ΔH}\right)}^{\text{2}}}{\text{L}}$$ (19)

where K’ is the surface roughness factor; Kr is the soil ridge roughness, calculated by Smith-Carson equation, unit cm; Crr is the random roughness factor, take 0, unit cm; L is the topographic relief parameter, △H is the difference in elevation within the distance L, △H is realised by using the focal statistics tool in ArcGIS software, L takes 1km..

#### Calculation of retention rate of WEPS.

In order to more accurately quantify the retention rate of the windbreak service function and to avoid the influence of climatic factors on the amount of windbreak service function, the F-value is calculated as follows:


$$\text{F}=\frac{\text{WEPS}}{\text{SLR}}\times \text{100\%}$$ (20)

where F is the retention rate of the wind and sand control service function.

### Geodetector

The Geodetector was mainly used to analyse the correlation of the variable Y with the selected eight X factors and their interactions. This is because the geo-detector q values have clear physical meaning with no assumption oflinearity, allowing us to objectively show that the dependent variable explains 100 q% of the difference. This study applied factor detectors and interaction detectors ingeo-detectors to analyze the correlations between the SL and selected impact factorsmore comprehensively, The formula used to calculate for the value of q was


$$\text{q}=1-\frac{\sum_{\text{h}=\text{1}}^{\text{L}}{\text{Nhσ}}_{\text{h}}^{\text{2}}}{\text{N}\sigma^{\text{2}}}=\text{1}-\frac{\text{SSW}}{\text{SST}}$$ (21)

where h = 1, 2,...,L is the stratification of variable Y or factor X, that is, the classification orpartition; Nh and N are the number of units in the laver and the whole area, respectively; ϭh2 and ϭ2 are the variance of Y with in the layer and in the whole area, respectively; and SSW and SST are sum of the variances within the layer and the total variance of the whole area, respectively.

Risk area detection was used to judge whether there was a significant difference in the average Y between the sub-areas of two influencing factors.


$$\text{t}=\frac{\bar{\text{Y}}\text{h}=\text{1}-\bar{\text{Y}}\text{h}=\text{2}}{{\left[\frac{\text{Var(}\bar{\text{Y}}\text{h}=\text{1)}}{\text{nh}=\text{1}}�\frac{\text{Var(}\bar{\text{Y}}\text{h}=\text{2)}}{\text{nh}=\text{2}}\right]}^{\frac{\text{1}}{2}}}$$ (22)

where Yh is the average value of the sub-areas h, nh is the numbers of samples in the sub-areas (h), and Var represents the variance.

Ecological detection was used to evaluate whether the influences of two influencing factors, X1 and X2, on the spatial differentiation of the windbreak and sand fixation were significant. This was done using the F statistic:


$$\text{F}=\frac{\text{NX1}�\text{NX2}-\text{1}�\text{SSWX1}}{\text{NX2}�\text{NX1}-\text{1}�\text{SSWX2}}=\frac{\text{NX1}�\text{NX2}-\text{1}�\sum_{\text{h}=\text{1}}^{\text{L1}}{\text{Nhσ}}_{\text{h}}^{\text{2}}}{\text{NX2}�\text{NX1}-\text{1}�\sum_{\text{h}=\text{1}}^{\text{L2}}{\text{Nhσ}}_{\text{h}}^{\text{2}}}$$ (23)

where NX1, and Nx2 are the sample numbers of impact factors X1 and X2, respectively; SSWX1 and SSWX2 represent the sum of the variance within the layers corresponding to theimpact factor stratification; and L1 and L2 represent the number of layers in impact factors X1 and X2.

In order to assess whether the explanatory power of the dependent variable increases when the influencing factors work together, it is necessary to select the dominant factor. The interaction detector is used to identify the interaction between variables to evaluate the explanatory power of the joint and independent effects of each explanatory variable on the response variable.

**Fig 2:**
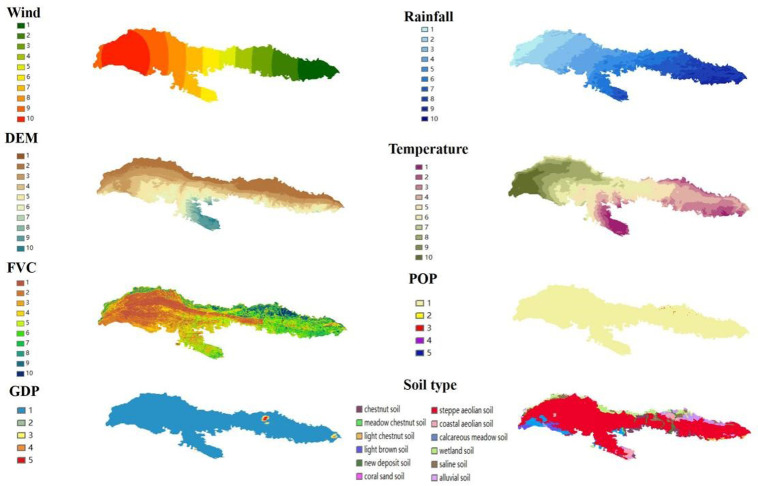
Input factors detected in the Kubuqi Desert.

Changes in SL are simultaneously influenced by natural factors such as climate, soil and vegetation types, as well as human activities. In view of this, six natural factors and two socio-economic factors were selected as detection factors in this study to analyse the drivers of annual mean WEPS changes in the Kubuqi Desert from 2000 to 2022. Based on data types and operability, the soil types of the Kubuqi Desert were classified into 14 categories. In addition, the number of variables categorised in this study partly refer to the categorisation in previous study [27]. For example, population density and GDP per unit area were classified into 5 categories, and the rest of the variables were classified into 10 categories. (Fig 2).

### Data source

All the data used in this paper were shown in Table 1, all the aforementioned data were resampled to a spatial resolution of 1km.

**Table 1:** Relevant data used in this study.

Factors	Input parameters	Data types	Data sources
	Wind speed	Average daily	http://cdc.cma.gov.cn
	Temperature	Average daily	http://cdc.cma.gov.cn
Weather factor	Precipitation	Average daily	http://cdc.cma.gov.cn
	Sunshine duration	Average daily	http://cdc.cma.gov.cn
	Snow depth	Average daily	http://data.tpdc.ac.cn
	Potential evapotranspiration	Monthly	https://data.tpdc.ac.cn/zh-hans/data/8b11da09-1a40-4014-bd3d-2b86e6dccad4
	Soil sand content	Constant type	https://www.ncdc.ac.cn/portal/metadata
Soil crust and	Soil silt content	Constant type	https://www.ncdc.ac.cn/portal/metadata
Erodible factor	Soil clay content	Constant type	https://www.ncdc.ac.cn/portal/metadata
	Soil organic matter content	Constant type	https://www.ncdc.ac.cn/portal/metadata
Roughness factor	Digital elevation model	Constant type	https://www.gscloud.cn/
Vegetation factor	Normalized difference vegetation index	Constant type	https://search.earthdata.nasa.gov/search
	land use types	Categorical type	https://zenodo.org/records/8176941
Other factors	Accuracy of the population	Constant type	https://landscan.ornl.gov
	Gross domestic product	Constant type	https://www.stats.gov.cn/

### Validation of RWEQ model accuracy

When RWEQ is applied to the Kubuqi Desert, it will be necessary to validate model accuracy. Short-term observations of wind erosion have been implemented to adapt RWEQ to the Kubuqi Desert. Field observations of wind erosion were implemented in the Kubuqi Desert from April to May 2023, and the observations were used to calibrate the parameters and simulation results. Ten observation siteswere deployed in the Kubuqi Desert, including four mobile dune sites and six farmland sites(Table 2). Horizontal sand fluxes were collected by a 0–100 cm sand collector. Each observation lasted for more than 30 min and the collected sediments were brought to the laboratory for weighing and calculation of wind erosion rates.

**Table 2:** Index of the probe factor.

Type of observation site	Location	Measured soil loss (kg/m^2^)	Predicted soil loss (kg/m^2^)	Expression of the fitting line	R^2^	RMSE
Mobile dunes 1 Mobile dunes 2 Mobile dunes 3 Mobile dunes 4	108.26E, 40.71N	5.30	10.26	y=0.54x+1.07	0.52	1.56
	108.29E, 40.70N	13.97	14.62			
	108.35E, 40.70N	4.32	1.27			
	108.29E, 40.71N	10.48	12.65			
Farmland 1 Farmland 2 Farmland 3 Farmland 4 Farmland 5 Farmland 6	109.95E, 40.30N	5.87	6.02	y=1.3x+0.11	0.70	0.99
	109.90E, 40.31N	6.45	6.76			
	109.82E, 40.37N	5.36	11.31			
	108.37E, 40.70N	1.54	1.42			
	108.36E, 40.70N	0.11	0.10			
	108.41E, 40.71N	0.03	0.28			

In this study, we selected the R^2^ and RMSE as two key parameters to evaluate the accuracy of the RWEQ model. We applied an accuracy criterion of ±50% of the observed values. Among all the wind erosion events, six were accurately estimated, three were overestimated, and one was underestimated. The RMSE values were smaller for the farmland and larger for the dunes. By analyzing the fitting curves of the simulated and measured values for both farmland and dunes, and considering the R^2^ values ranging from 0.52 to 0.7, we concluded that the model’s accuracy is suitable for simulating the amount of soil wind erosion in the farmland of the Kubuqi Desert.

## Results

### Spatiotemporal distribution characteristics of five factors in the study area

**Fig 3:**
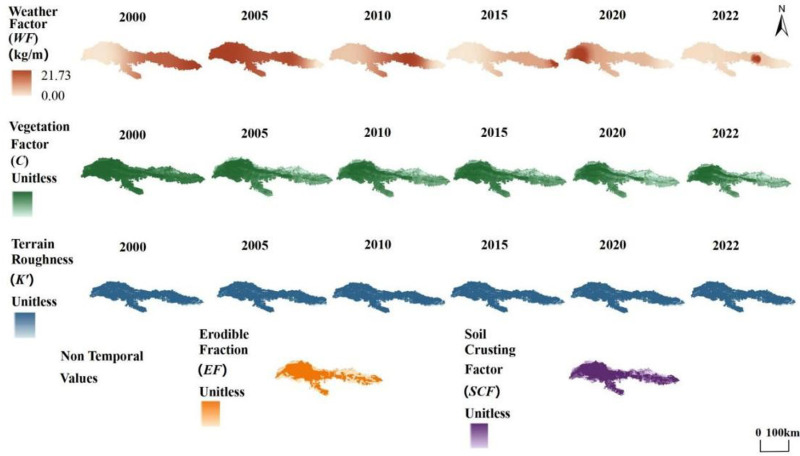
The five factors of the of the Kubuqi Desert from 2000 to 2022.

The main factors of the RWEQ are the weather factor (WF), the vegetation factor (C), the surface terrain roughness factor (K’), the erodibility factor (EF), and the soil crust factor (SCF). The weather factor (WF) represents the effect of climate on wind erosion and combines wind speed, soil moisture, and snow cover. As shown in Fig 3, The WF factor was highest in 2005 at 21.73 kg/m. Increases in soil moisture, vegetation, and snow cover reduced the sensitivity to wind. Therefore, these factors must be low for wind to cause severe soil losses. K’ behaved more similarly in different years, while there was no yearly variation in SCF and EF in the area, and non-time-varying soil composition data were used in this study.

### Characteristics of spatial distribution of SL, SLR, F and WEPS in the study area

**Fig 4:**
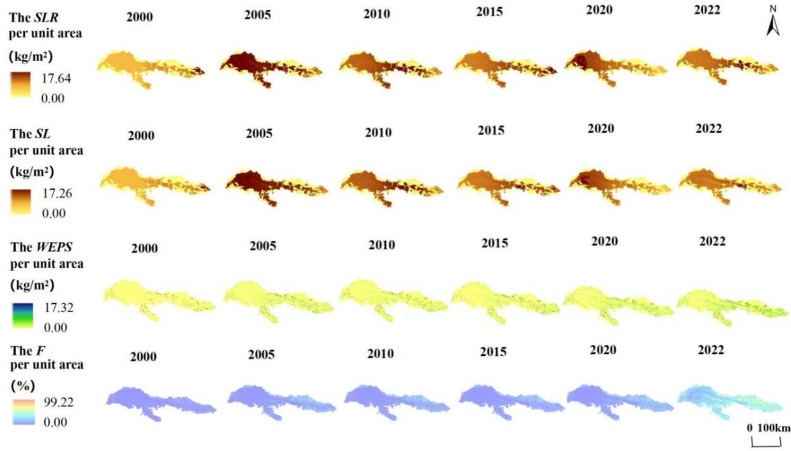
The amount of SLR, SL, WEPS and F per unit area of the Kubuqi Desert from 2000 to 2022.

From 2000 to 2022, the total SLR (potential wind erosion) of the Kubuqi Desert ranges from 8.35×10^7^t to 19.73×10^7^t, and the average SL (actual wind erosion) per unit area ranges from 4.49 to 10.61 kg/m^2^. The SLR values per unit area were calculated from 0 to 17.64 kg/m^2^ (Fig 4), with higher SLR values per unit area in the west and lower values in the east, and showing the trend of ‘increasing-decreasing-decreasing-increasing-decreasing’. This indicates that the western part of the Kubuqi Desert, including the desert area, is more vulnerable to the threat of wind erosion.

The total SL (actual wind erosion) of the Kubuqi Desert was calculated in the range of 8.24×10^7^t to 18.99×10^7^t, and the average SL (actual wind erosion) per unit area was calculated in the range of 4.43 to 10.21 kg/m^2^. The value of SLR per unit area ranged from 0 to 17.26 kg/m^2^, reaching a maximum in 2005 and decreasing continuously between 2005 and 2015. The SL value per unit area was higher in the west and lower in the east. This indicates that strong wind erosion occurs in the western part of the Kubuqi Desert. In addition, comparing the SL and SLR values in the eastern region, it can be seen that the vegetation cover significantly reduces the occurrence of wind erosion.

Between 2000 and 2022, the total WEPS ranges from 0.35×10^7^ t to 1.26×10^7^ t, The average WEPS per unit area is calculated in the range of 0.19 to 0.68 kg/m^2^. WEPS per unit area decreased by 1.5%, 21.34% and 26.89% in the time periods 2005–2010, 2010–2015 and 2020–2022, which increased in the rest of the time period. The calculated WEPS per unit area ranged from 7.71 to 17.32 kg/m^2^, and the change of WEPS over time showed the same trend as the SLR value, which was ‘increasing-decreasing-decreasing-increasing-decreasing’. The WEPS per unit area was the lowest in 2000 and the highest in 2005, and the overall WEPS per unit area showed a distribution pattern of high in the east and low in the west.

The F-value was calculated in the range of 0~ 99.22%, with higher F-value in the east and lower F in the west, and the average WEPS per unit area was calculated in the range of 4.34~ 9.44%. The F per unit area ranged from 42.58 ~ 49.61% and decreased by 0.01% and 14.17% in the time periods 2005–2010 and 2020–2022, which increased in the rest of the time period. Unlike the trend of WEPS over time, F shows a ‘rising-falling-rising-rising-falling’ pattern over time. The areas with higher retention rates of wind and sand services are mainly distributed in the eastern region with better vegetation cover, with a more obvious geographical distribution from west to east. This shows that the ecosystem in the eastern part of the Kubuqi Desert contributes more to the prevention of wind erosion.

### Changes in SL, SLR, F and WEPS spatial patterns in the Kubuqi Desert

**Fig 5:**
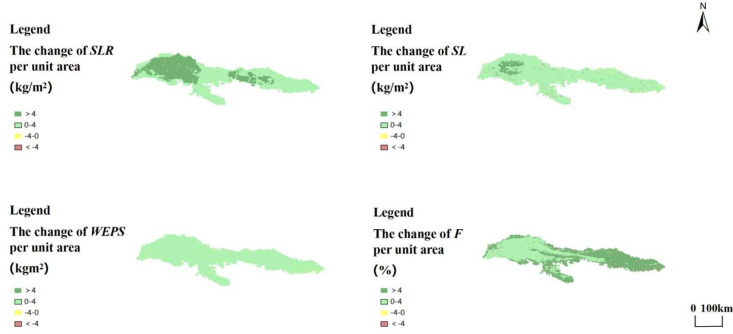
Changes in SL, SLR, WEPS and F per unit area of the Kubuqi Desert from 2000 to 2022.

As illustrated in Fig 5, between the years 2000 and 2022, the four factors of SLR, SL, WEPS and F exhibit a greater extent of increasing regions compared to those that are decreasing. The SLR per unit area in the Kubuqi Desert varies from 0.01 to 4.21 kg/m^2^, and the area with more increase in SLR per unit area mainly occurs in the west, with an average change of 1.62 kg/m^2^.The SL per unit area varies from -6.97 to 5.54 kg/m^2^, and the area with more increase in SLR per unit area also mainly occurs in the west, but the area with less than the SLR increased to the same extent in less area, with an average change of 1.97 kg/m^2^.

The WEPS per unit area in the Kubuqi Desert varied from -1.41 to 2.41 kg/m^2^, with an average change of 2.01 kg/m^2^ The decrease in WEPS per unit area was mainly concentrated in the central and eastern parts of the desert, with a sporadic distribution. The changes in F per unit area ranged from -37 to 15.31 per cent, and the average change in F was 1.55 per cent. The areas with more increase in SLR per unit area were opposite to SLR and SL, which were mainly in the east, and the areas with decrease were also mainly concentrated there, with a sporadic distribution.

### Interaction analysis of driving factors of soil wind erosion spatial pattern in the Kubuqi Desert

In this study, the annual average SL values per unit area from 2000 to 2022 were scored as Y-factors. The intensity of soil wind erosion is often influenced by natural (climate and vegetation cover) as well as human-driven (with economic, social and anthropogenic attributes) factors. Based on the process of calculating soil wind erosion and considering the actual environment of the study area, the average annual wind speed, average annual precipitation, DEM, average annual temperature, average annual FVC, average annual population density, real gross domestic product GDP, and soil category were set as X1-X8 coefficients. In this context, X1 through X5, along with X8, represent selected natural drivers, while X6 and X7 denote anthropogenic factors.

**Fig 6:**
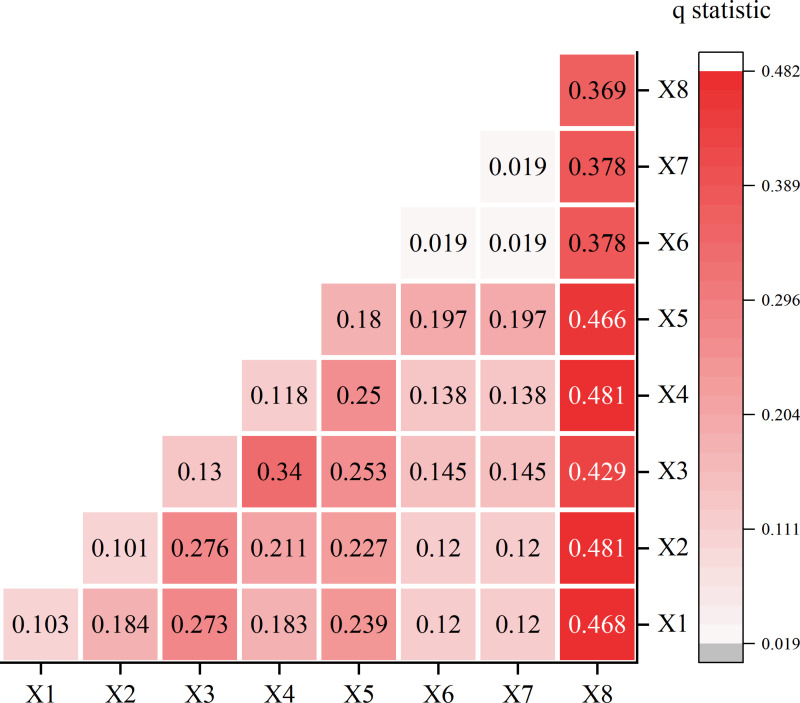
Geographic detection interaction diagram.

Since the geodetector requires the independent variable X to be discrete, the continuous variables need to be discretised first. In this study, the GD package, which is based on Excel software, was utilised to be able to optimise the geodetector data easily and quickly, and the following data for each factor is shown below (Fig 6).

The main influencing factors of SL in the Kubuqi Desert and their explanatory power q-values (Table 3) were obtained by geoprobes, and the factors have different degrees of influence on SL with obvious variability, and the explanatory power values in order from largest to smallest are as follows: soil type (0.369)> annual vegetation cover (0.180)> elevation (0.130)> average annual temperature (0.118)> average annual wind speed (0.103)> mean annual precipitation (0.101)> population size (0.019) = GDP (0.019). Soil type has the highest degree of influence on SL and becomes the dominant factor influencing the amount of wind erosion in the area; vegetation cover, elevation, mean annual temperature, mean annual wind speed, and mean annual precipitation have a degree of influence greater than 0.1 on SL and become the main factors; population size and GDP have a lesser degree of influence and are the secondary driving factors.

**Table 3:** Contribution of various factors to SL.

Influencing factor	Influence (q)	Ordination
Soil type (X8)	0.369	1
Annual FVC (X5)	0.180	2
DEM (X3)	0.130	3
Average Annual temperature (X4)	0.118	4
Average Annual Wind speed (X1)	0.103	5
Average Annual rainfall (X2)	0.101	6
POP (X6)	0.019	7
GDP (X7)	0.019	8

The results of the test factor (Table 4) showed that factor X3 DEM, factor X5 average annual FVC, and average annual X8 Soil category were significantly different from all other influencing factors, while factor X6 Population size, and factor X7 Gross Domestic Product were not significantly different from all other influencing factors.

**Table 4:** Statistical significance between detecting factors.

Detecting factor	X1	X2	X3	X4	X5	X6	X7	X8
X1								
X2	N							
X3	Y	Y						
X4	Y	Y	N					
X5	Y	Y	Y	Y				
X6	N	N	N	N	N			
X7	N	N	N	N	N	N		
X8	Y	Y	Y	Y	Y	Y	Y	

Notes:

Y indicates a statistically significant difference (95% confidence level) between the two factors; N indicates no significant difference.

After the identification of single factors, this study also explored the interaction between factors to identify whether the joint effect of different influencing factors increases or decreases the explanatory power of soil wind erosion, and the results of the multi-year average interaction detector showed that the strength of the influence of factors was significantly increased after the interaction, which was a non-linear enhancement relationship, indicating that the influences of each factor on SL are interrelated and synergistic. Soil wind erosion was most strongly influenced by the interaction of X8 soil type with X4 mean annual air temperature and X2 mean annual precipitation, which could explain 48.1% of the soil wind erosion in the study area; the stronger interaction was between X1 mean annual wind speed and X8 soil type, which could explain 46.8% of the soil wind erosion in the study area, followed by mean annual vegetation cover, which could explain 46.6% of the soil wind erosion in the study area, which further indicates that the interaction of weather factor. While X8 soil type interacted with all other factors by more than 30%, indicating that this factor plays an important role in preventing soil wind erosion. In addition, when X5 annual average vegetation cover interacted with factors other than X6 population and X7 gross domestic product at X6 was greater than 20%, indicating that the influence of this factor on the change of WEPS could not be ignored. The above results indicate that the interaction of weather factor, vegetation factor and soil factor is the dominant factor affecting the amount of soil wind erosion in the Kubuqi Desert.

## Discussion

### Causes and spatiotemporal differentiation of WEPS

The pattern of WEPS changes in the Kubuqi Desert during the 22-year period is similar to that of the SLR, mainly because WEPS depends mainly on natural factors such as wind and precipitation. Combined with the results of the geoprobe analyses, it can be concluded that the causes of the above changes in the Kubuqi Desert from 2000 to 2022 are mainly related to the changes in the spatial and temporal patterns of vegetation cover during this period. With the implementation of a series of ecological protection measures, such as fencing and sealing, returning ploughland to forests and so on, it effectively promotes the restoration of vegetation cover, so that the sand ecosystem can be restored, which in turn improves the ability of the Kubuqi Desert in preventing winds and fixing sands.

**Fig 7:**
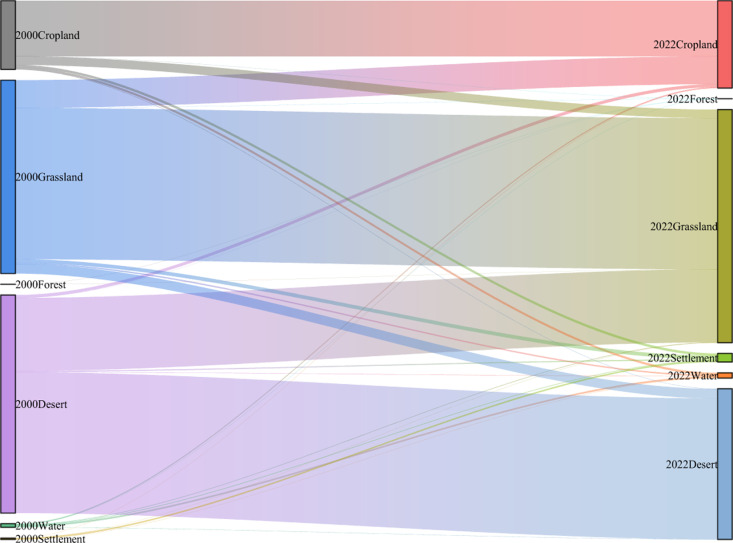
The area changes in ecosystems in the Kubuqi Desert from 2000 to 2022.

In order to analyse the effect of land use type change on soil wind erosion control WEPS from 2000 to 2022, two land use data (2000 and 2022) at the starting time point of the study period were selected for the land use change identified by WSFS drivers, given the availability of data. In this paper, changes in different land use types are analysed, and the results show that the conversion area of desert ecosystems to grassland ecosystems is the highest during the 22-year period, followed by the conversion area of grassland to cropland, and the increase in the area of forest ecosystems is mainly due to the conversion of cropland and watersheds (Fig 7). Among the different types of land use changes, an increase in the area of forest ecosystems leads to an increase in WEPS, while an increase in the area of desert ecosystems leads to a decrease in the average WEPS in the Kubuqi Desert region. Land use/cover changes (e.g., agricultural land restoration to forests, shrubs, and grasslands) play a key role in reducing soil wind erosion modulus and enhancing regional WEPS. It was discovered that the expansion of grassland resulting from large-scale ecological initiatives, including the conversion of farmland back into grassland, contributed to the enhancement of WEPS.

However, unsuitable land use can lead to an increase in the area of deserts, which is the main reason for the decrease in the total amount of WEPS. Therefore, when implementing ecological projects, attention should be paid not only to severely desertified areas but also to grasslands with degradation trends to prevent further deterioration of low-cover grasslands. Soil wind erosion can be prevented by targeting rational land use control and territorial spatial planning, and accelerated economic development can directly or indirectly improve the incidence of soil erosion by wind.

Simultaneously, during urban sprawl, there frequently arises a clash between human requirements and the judicious management and exploitation of land resources. This often leads to the transformation of wind-sheltered, high-quality land uses—such as farmland, forests, and grasslands—into developed areas. Nevertheless, developed land does not experience the processes of soil wind erosion or sand stabilization, resulting in a reduction of WEPS.

### Weaknesses and prospects

In previous studies, the weather factor and vegetation factor plays the most important role in preventing soil wind erosion among the factors [28], which is consistent with the results of this paper. However, interestingly, the interaction analysis in this paper shows that in the Kubuqi Desert region, the interaction between the soil factor and the two dominates the soil wind erosion in the region, which is a point of difference from the previous studies and may be due to the more complex soil types in the region.In addition, regarding the RWEQ model in this paper, there is a problem of discrepancy between measured and simulated values, and Van Peltalso reported a similar problem [29], so further calibration of the modelmay be a trend in the future application of the RWEQ model in the study of wind erosion estimation in China [30]. So further in-depth studies are needed to investigate how the interaction between more than two factors affects the WEPS capacity and spatial and temporal variations, and whether the neighbouring areas have an impact on the wind and sand fixation services in the Kubuqi Desert.

To ensure the long-term maintenance of desert ecological construction, it is imperative to establish a stable investment mechanism. We must also actively foster the economic valuation of desert ecological products, alongside traditional forestry, agricultural, and livestock production. This includes integrating emerging industries such as photovoltaic power generation and aquaculture [31], as well as developing eco-tourism and recreational activities. These measures will not only stimulate economic growth but also offer a more sustainable solution for the conservation of desert ecosystems. Furthermore, these initiatives will provide consistent financial backing for the preservation and restoration of desert ecological integrity.

In addition, the Kubuqi Desert is a vast area with diverse geographic environments, coupled with marked differences in human activities and resource use in different regions, so it is recommended that a zoning management strategy be implemented for the Kubuqi Desert, in order to better take into account the differences in ecosystems and to strengthen the protection of vulnerable areas in a targeted manner, so as to improve the overall efficiency of desertification management. With the development of the economy, it is possible to indirectly assist in sand control strategies (such as crop rotation and grazing), and it is also necessary for the Government to formulate more precise, efficient, scientific and rational agricultural and livestock production policies, and to better resolve and deal with the contradictions between the ‘three livelihoods’ (i.e., production, life and ecology).

## Conclusions

In this study, the RWEQ model was used to synthesise the annual SL, annual SLR, annual WEPS and annual F data of the Kubuqi Desert region from 2000 to 2022, and the spatial and temporal distribution characteristics of the four were discussed, and then the spatial distribution characteristics of SL and its changing law during the 22-year period in the Kubuqi Desert region were explored, and the geoprospector was used to quantitatively analyse the relationship between the six natural factors and the two human activity factors driving SL in the Kubuqi Desert region, and drew the following key conclusions:

Overall, the degree of soil wind erosion in the Kubuqi Desert region shows a distribution pattern of ‘high in the west and low in the east’, with a high spatial distribution of WEPS in the east and a low spatial distribution in the west, and the trend of SLR and WEPS over time is the same, which shows a trend of ‘rising-declining-declining-rising-declining’. SLR and WEPS have the same trend over time, showing the trend of ‘rising - falling - falling - rising - falling’. The F-value is higher in the eastern region and lower in the western region.

Among the eight driving factors of SL in the Kubuqi Desert, soil type is the most important driver, vegetation cover, elevation, mean annual temperature, mean annual wind speed and mean annual precipitation, are the main driving factors, and population size and GDP are the secondary driving factors. The interaction analysis showed that the interaction of weather factor, vegetation factor and soil factor is the dominant factor influencing the amount of soil wind erosion in the Kubuqi Desert.

The implementation of ecological projects such as the Three-North Protective Forest Project and returning farmland to forest increased WEPS by increasing the forest area. unsui /////// land use can lead to an increase in desert ecosystems leading to a decrease in WEPS in the Kubuqi Desert.
